# Curcumol inhibits encephalomyocarditis virus by promoting IFN-β secretion

**DOI:** 10.1186/s12917-021-03015-4

**Published:** 2021-09-30

**Authors:** Jiangang Zheng, Yinlan Xu, Ajab Khan, Panpan Sun, Yaogui Sun, Kuohai Fan, Wei Yin, Shaoyu Wang, Hongquan Li, Na Sun

**Affiliations:** 1 College of Veterinary Medicine, Shanxi Agricultural University, Taiyuan, Shanxi 030000 P.R. China; 2 Laboratory Animal Center, Shanxi Agricultural University, Taiyuan, Shanxi 030000 P.R. China; 3 School of Community Health, Faculty of Science, Charles Sturt University, Bathurst, New South Wales 2800 Australia

**Keywords:** EMCV, Curcumol, IFN-β, Antivirus

## Abstract

**Background:**

Encephalomyocarditis virus (EMCV) infection can cause reproductive failure in sows and acute myocarditis and sudden death in piglets. It has caused huge economic losses to the global pig industry and that is why it is necessary to develop effective new treatment compounds. Zedoary turmeric oil has been used for treating myocarditis. Curcumol extracted from the roots of curcuma is one of the main active ingredient of zedoary turmeric oil. The anti-EMCV activity of curcumol along with the molecular mechanisms involved with a focus on IFN-β signaling pathway was investigated in this study.

**Method:**

3-(4,5-dimethyithiazol-2-yl)-2,5-diphenyltetrazolium bromide (MTT) assay was used to determine the maximum non-toxic concentration (MNTC), 50% cytotoxic concentration (CC_50_), maximum inhibition rate (MIR) and 50% effective concentration (EC_50_) against EMCV. Through EMCV load, the anti-viral effect of curcumol was quantitatively determined using real-time quantitative PCR (qPCR). The effect of curcumol on the expression of IFN-β was investigated using real-time quantitative PCR and ELISA. Western blot was used to determine the amounts of MDA5, MAVS, TANK, IRF3 and P-IRF3 proteins in human embryonic kidney 293 T (HEK-293 T) cells infected with EMCV.

**Results:**

The results of MTT showed that compared with the ribavirin positive control group, the maximum inhibition ratio (MIR) of curcumol was greater but the selection index (SI) value was much smaller than that of ribavirin. The results of qPCR showed that curcumol and ribavirin significantly reduced the replication of EMCV in HEK-293 T cells. The curcumol (0.025 mg/mL) treatment has significantly increased IFN-β mRNA expression in the EMCV-infected HEK-293 T cells while ribavirin treatment did not. The results of ELISA showed that curcumol (0.025 mg/mL and 0.0125 mg/mL) has significantly increased the expression of IFN-β protein in EMCV-infected HEK-293 T cells. The results of Western blot showed that curcumol can inhibit the degradation of TANK protein mediated by EMCV and promote the expression of MDA5 and P-IRF3, while the protein expression level of MAVS and IRF3 remain unchanged.

**Conclusion:**

Curcumol has biological activity against EMCV which we suggest that IFN-β signaling pathway is one of its mechanisms.

**Supplementary Information:**

The online version contains supplementary material available at 10.1186/s12917-021-03015-4.

## Background

Interferon is a glycoprotein induced by viral infection [1], bacteria and protozoa [2]. It was also shown that microbial nucleic acids, lipids, polysaccharides or proteins trigger induction of IFNs through activation of TLRs [3]. An early pivotal discovery identified double-stranded (ds) RNAs as potent inducers [4]. Three types of IFN families are known type I (IFN-1, mainly IFN-α/β), type II (IFN-II or IFN-γ) and type III (IFN-III or IFN-λ) [5]. Interferon acts via autocrine and paracrine modes to induce the antiviral response in host cells and also in neighboring cells containing interferon receptors. IFN-I subspecies activate a common type I IFN receptor (IFNAR) which sends a signal to the nucleus. The STAT proteins are latent cytoplasmic transcription factors which become phosphorylated by the Janus kinases-1 (JAK-1) and human tyrosine kinase-2 (TYK-2). Phosphorylated signal transducer and activator of transcription-1 (STAT-1) and signal transducer and activator of transcription-2 (STAT-2) recruit a third factor, IRF-9, to form a complex known as IFN-stimulated gene factor 3 (ISGF-3) which translocates to the nucleus and binds to the IFN-stimulated response element (ISRE) in the promoter region of interferon stimulated genes (ISGs). Type I IFNs activate the expression of several hundred ISGs [6], such as 2’-5’ oligoadenylates synthesis (OASs) [7], double-stranded RNA-dependent protein kinase (PKR) [8], and myxo-virus resistance protein (MX) [9]. These antiviral proteins can inhibit viral replication. The defense mechanism established by IFN can also have effects on cancer cells [10, 11] and inflammation [12]. In particular, the type I interferon has indirect antiviral effect. The rapid production of type I interferon is an important factor in the early stages of the host’s response to the viral infection [13–15].

EMCV belongs to the *Picornaviridae* family and is a nonencapsulated single-stranded RNA virus. EMCV infection can cause reproductive failure in sows and acute myocarditis and sudden death in piglets [16–18]. Studies have shown that melanoma differentiation-associated protein-5 (MDA5) is a cytoplasmic virus sensor that recognizes double-stranded RNA (dsRNA) produced during EMCV replication [19, 20] and activates Tank-binding kinase-1 (TBK1) and IKB kinase-ε (IKK-ε) through mitochondrial antiviral signaling protein (MAVS) [21, 22]. These two activated kinases can phosphorylate interferon regulatory factor-3 (IRF3), and the phosphorylated IRF3 is then transferred from the cytoplasm to the nucleus, promoting the expression of type I interferon [23, 24].

TRAF family member-associated NF-kB activator (TANK) was originally discovered to be a TRAF-binding protein, which can inhibit TNF receptor associated factor-2 (TRAF2)-mediated activation of nuclear transcription factor-kB (NF-kB) in the tumor necrosis factor-α (TNF-α) and CD-40 signaling pathways [25]. A previous study revealed that TANK, TRAF2 and TBK1 form a trimer to activate NF-kB [26]. Studies have shown that TANK can link TBK1 and IKK-ε to synergistically active the NF-kB [27].

Previous data have indicated that zedoary turmeric oil exhibits a wide array of therapeutic activities, such as inhibits the growth of many viruses and bacteria like Staphylococcus aureus, β-hemolytic streptococcus, respiratory syncytial virus [28] and influenza virus [29]. Zedoary turmeric oil also depresses the growth of tumor cells, especially for hepatoma cells [30]. Curcumol extracted from the roots of Curcuma is one of the main active ingredient of zedoary turmeric oil [31, 32]. EMCV is specifically recognized by MDA5 and mediates the production of IFN-β. Li et al. have shown that EMCV 3C protease can hydrolyze TANK and interfere with TANK-TBK1-IKK-ε-IRF3 complex formation, thereby inhibiting the production of type I interferon [33]. Therefore, we investigate whether curcumol can reduce virus replication in EMCV-infected HEK-293 T cells. Further in vitro experiments were used to determine whether MDA5/IRF3/ IFN-β signaling pathway is involved in the anti-EMCV activity of the curcumol.

## Results

### Cytopathic effects

**Fig. 1:**
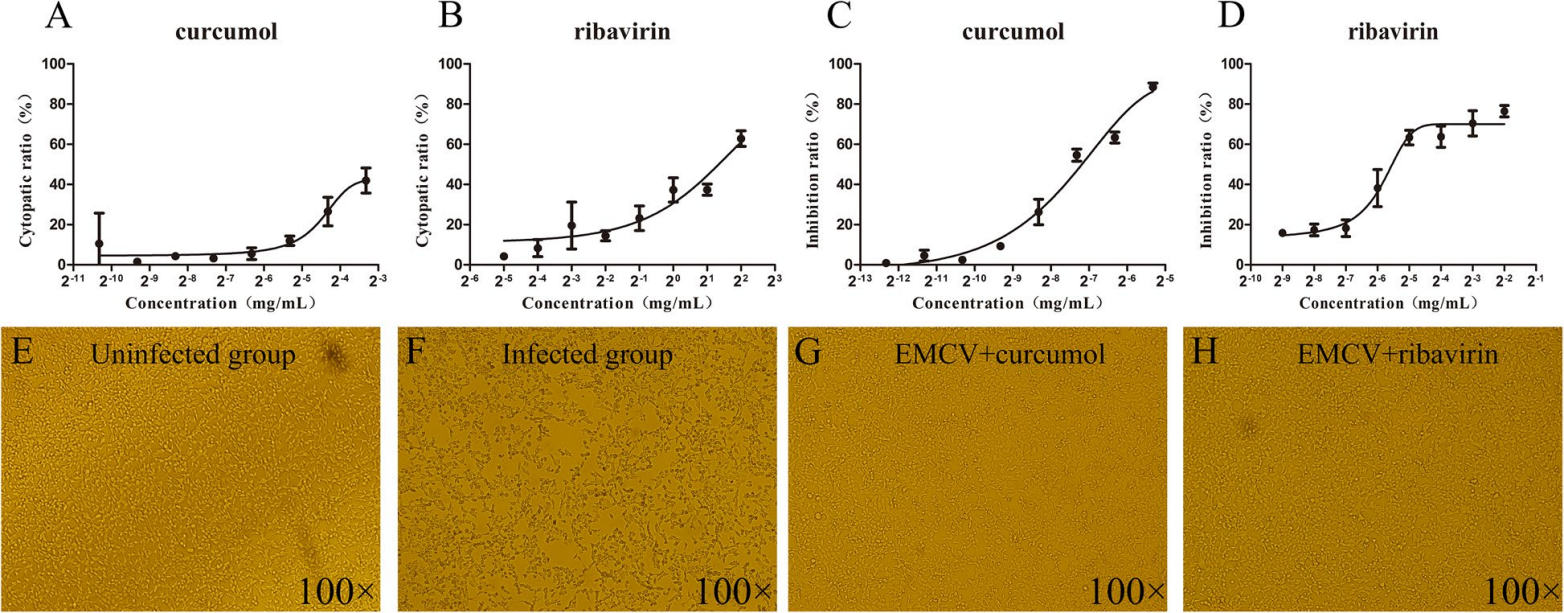
Cytotoxicity and antiviral activities of tested compounds. **A**, **B** HEK-293 T cells treated with different concentrations of curcumol and ribavirin for 72 h, and the cytopathic rate curve measured by MTT method. **C**, **D** Curcumol and ribavirin were selected to treat EMCV-infected HEK-293 T cells for 24 h, and the compounds inhibition rate curve measured by MTT method. **E**–**H** The morphology of HEK-293 T cells post 24 h of treatment. **E** Uninfected group, **F** Infected group, **G** Curcumol group, **H** Ribavirin group

The MNTC and CC_50_ of curcumol (Fig. 1A) and ribavirin (Fig. 1B) were determined using MTT assay. In a dose dependent manner, HEK-293 T cells showed different degrees of lesions, cell shrinkage, rupture, and cytopathic effect. The MNTC of curcumol (0.025 mg/mL) was less than the MNTC of ribavirin (0.25 mg/mL) and the CC_50_ value (0.124 mg/mL) was also less than that of ribavirin (2.538 mg/mL) (Table 1).

**Table 1:** The cytotoxicity and antiviral activity of the compounds used in the test (mean ± SD)

Constituents	Dissolution	MNTC^a^ (mg/mL)	CC_50_^b^ (mg/mL)	MIR^c^ (%)	EC_50_^b^ (mg/mL)	SI^d^
Curcumol	1% DMSO	0.025	0.124 ± 0.021	89	0.006 ± 0.0015	20
Ribavirin	DMEM	0.25	2.538 ± 0.54	80	0.023 ± 0.012	110

^a^MNTC means maximum non-toxic concentration

^b^CC_50_ and ^b^EC_50_ mean 50% cytotoxic concentration and 50% effective concentration

^c^MIR means maximum inhibition ratio

^d^SI meas selection index

### Anti-EMCV activity of curcumol

The MNTC of test compounds were selected to treat EMCV-infected HEK-293 T cells for 24 h. The results of MTT showed that compared with the ribavirin positive control group, the maximum inhibition ratio (MIR) of curcumol was greater but the selection index (SI) value was much smaller than that of ribavirin. The results showed that curcumol displayed MIR > 50% and SI > 3 and ribavirin showed an expected anti-EMCV activity (Table 1). Both curcumol (Fig. 1C) and ribavirin (Fig. 1D) alleviated EMCV cytopathic effects in a dose- dependent manner. Microscopically, the infected group (Fig. 1F) showed an increase in cell gap, cell shrinkage and patches of cell death compared with the uninfected group (Fig. 1E). The condition of cells in the curcumol and ribavirin treatment groups were consistent with the uninfected group as shown in Fig. 1G and H which reduced the cytopathic effect caused by virus and maintained the normal cell morphology.

### Curcumol reduces the viral load in EMCV-infected HEK-293 T cells

**Fig. 2:**
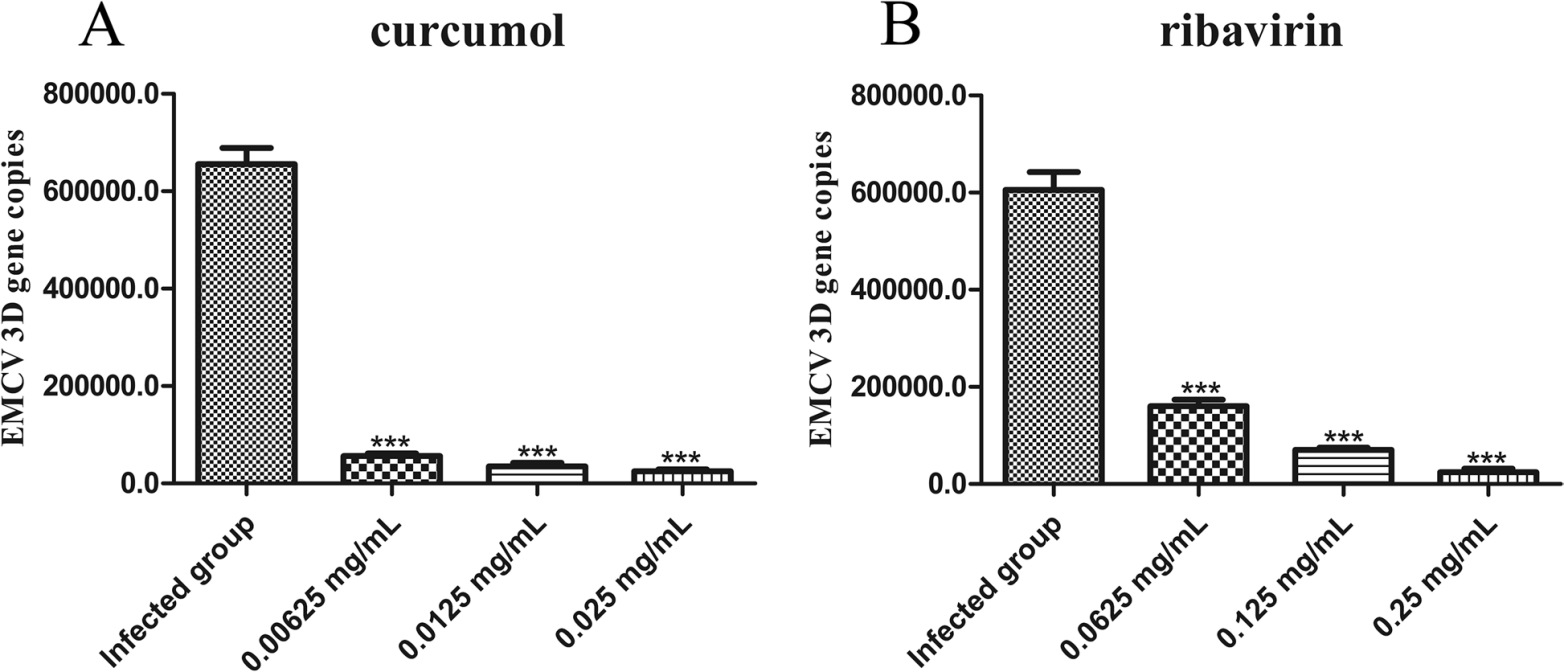
Anti-EMCV activities of curcumol and ribavirin detected by qPCR. **A**, **B** Cells were incubated with 100 TCID_50_/mL EMCV for 2 h, then curcumol (0.025, 0.0125 and 0.00625 mg/mL) and ribavirin (0.25, 0.125 and 0.0625 mg/mL) were added respectively and cultured for 24 h. The expression of the 3D gene was detected by qPCR (* *P* < 0.05, ** *P* < 0.01, *** *P* < 0.001)

Twenty-four hours after treating EMCV-infected HEK-293 T cells with curcumol and ribavirin, the viral load was calculated using qPCR to determine the anti-EMCV efficacy. Compared with the infected group, curcumol (Fig. 2A) and ribavirin (Fig. 2B) significantly (*P* < 0.001) reduced viral load. These results demonstrated that curcumol and ribavirin significantly reduced the replication of EMCV in HEK-293 T cells.

### Curcumol promotes the expression of IFN-β in EMCV-infected HEK-293 T cells

**Fig. 3:**
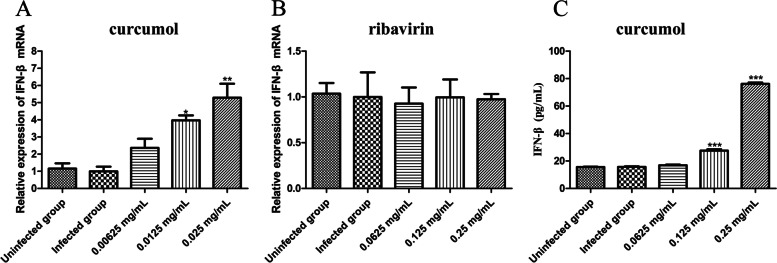
Effect of curcumol on the expression of IFN-β. **A**, **B** Curcumol (0.025, 0.0125 and 0.00625 mg/mL) and ribavirin (0.25, 0.125 and 0.0625 mg/mL) were selected to treat EMCV-infected HEK-293 T cells for 24 h, and the IFN-β mRNA measured by qPCR. **C** Curcumol (0.025, 0.0125 and 0.00625 mg/mL) were selected to treat EMCV- infected HEK-293 T cells for 24 h, and the IFN-β protein measured by ELISA (* *P* < 0.05, ** *P* < 0.01, *** *P* < 0.001)

Twenty-four hours after treating EMCV-infected HEK-293 T cells with curcumol and ribavirin, the expression of IFN-β mRNA was calculated using qPCR. There was no difference in IFN-β mRNA expression between the infected group and the uninfected group. Compared with the infected group, curcumol (0.025 mg/mL) treatment has significantly (*P* < 0.01) increased IFN-β mRNA expression in the EMCV-infected HEK-293 T cells (Fig. 3A) while ribavirin treatment did not (Fig. 3B). Twenty-four hours after treating EMCV-infected HEK-293 T cells with curcumol, the expression of IFN-β protein was calculated using ELISA. The curcumol (0.025 mg/mL and 0.0125 mg/mL) has significantly (*P* < 0.001) increased the expression of IFN-β protein in EMCV-infected HEK-293 T cells (Fig. 3C).

### Curcumol relieves the cleavage of TANK by EMCV and promotes the formation of IRF3 phosphorylation

**Fig. 4:**
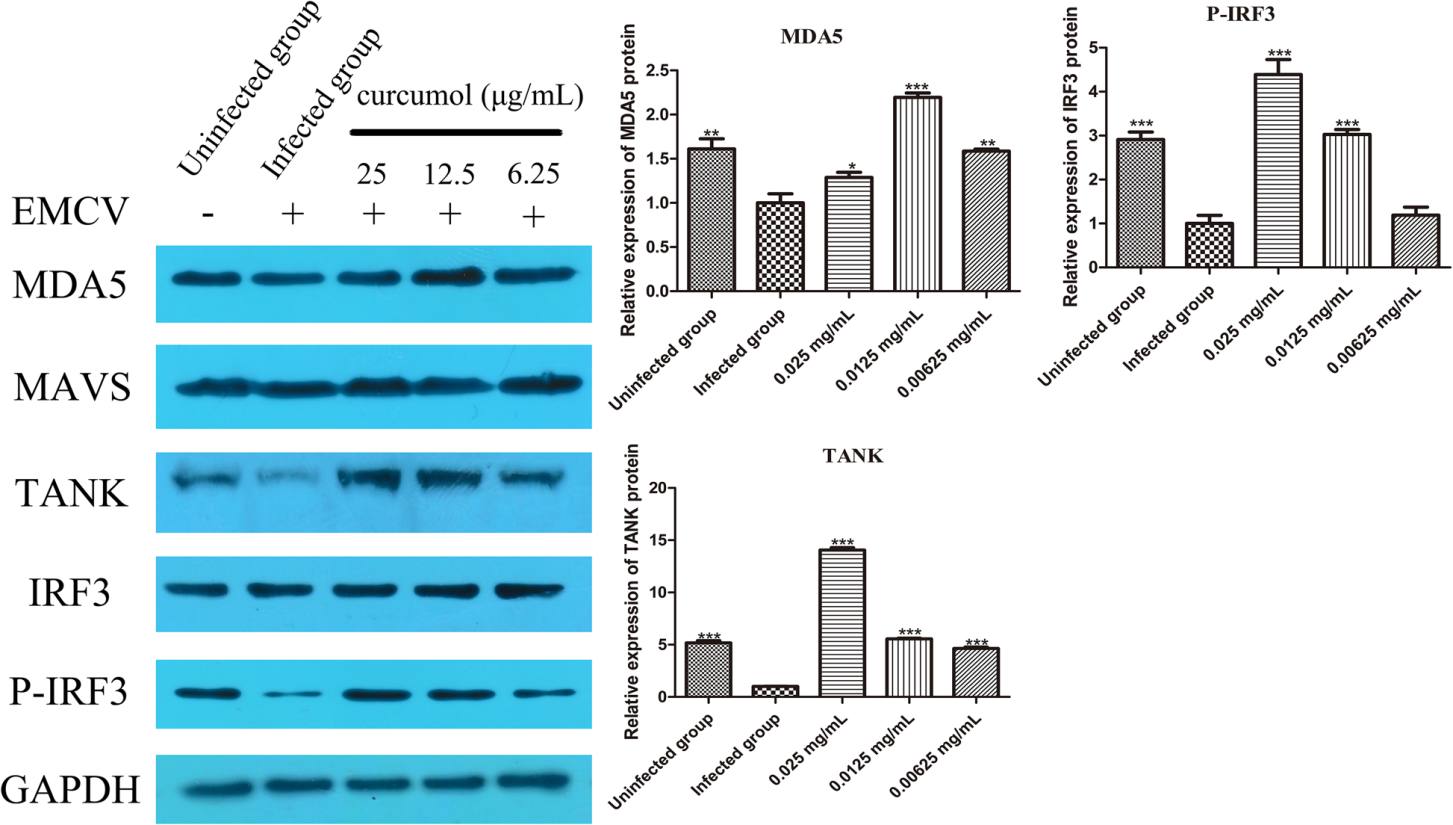
Curcumol (0.025, 0.0125 and 0.00625 mg/mL) and ribavirin (0.25, 0.125 and 0.0625 mg/mL) were selected to treat EMCV-infected HEK-293 T cells for 24 h, and the expression of MDA5, MAVS, TANK, IRF3, P-IRF3 protein were detected by Western blot. Densitometric values of protein bands were quantified by the Image J. Data were analyzed using GraphPad Prism™ software 5.0 (GraphPad Software, Inc. California, USA). One-way analysis of variance (ANOVA) followed by a Dunnett’s post-test was used to determine the difference between the groups. All groups are compared with EMCV-infected group (* *P* < 0.05, ** *P* < 0.01, *** *P* < 0.001)

The expression of MDA5, TANK and P-IRF3 protein decreased significantly (*P* < 0.01) after HEK-293 T cells infection with EMCV while the expression of MAVS and IRF3 protein showed no significant change. There was no significant change in the expression of MAVS after 24 h of curcumol treatment, indicating that the increase of IFN-β does not depend on MAVS. Compared with the infected group, the expression levels of MDA5 and TANK protein were significantly increased after curcumol treatment, and the expression of P-IRF3 was increased while IRF3 expression remained unchanged. These results indicate that curcumol promotes IFN-β expression, possibly by alleviating the hydrolysis of TANK protein by EMCV (Fig. 4).

## Discussion

The aim of this study was to investigate the anti-EMCV effects of curcumol and associated molecular mechanisms. Compared with the infected group, at MNTC (curcumol 0.025 mg/mL and ribavirin 0.25 mg/mL), curcumol and ribavirin reduce cell shrinkage and rupture. The results showed that compared with the infected group, the EMCV copy number was significantly (*P* < 0.001) reduced when treated with curcumol at 0.025, 0.0125 and 0.00625 mg/mL, ribavirin at 0.25, 0.125 and 0.0625 mg/mL. The results also indicate curcumol and ribavirin can reduce virus replication, but the anti-EMCV activity of curcumol is more potent than ribavirin.

In terms of the HEK-293 T cells response towards EMCV infections, this study has confirmed that viral infection causes decreased MDA5 expression level in the EMCV infected HEK-293 T cells. Compared with the infected group, the expression level of MDA5 protein was significantly increased when treated with curcumol at 0.0125 mg/mL. MDA5 belongs to the RIG-I-like pattern recognition receptor which recognizes dsRNA produced during EMCV replication and mediates the production of type I interferon [19, 20]. Li et al. [34] showed that EMCV 2C specifically inhibits the MDA5 mediated IFN-β signaling pathway by interacting with MDA5 and disrupting its normal function. It is noted that the expression of MAVS, the downstream protein of MDA5 was remained unchanged in curcumol treatment. Thus, the link between viral RNA sensor proteins, MAD5 to TANK/TBK1/TRAF3 complex is not affected by curcumol treatment.

The TANK forming complex with TBK1, TNF receptor associated factor 3 (TRAF3) and other proteins can phosphorylate IRF3 [22]. Increased level of TANK may also affect the pathway for the activation of NF-κB signaling pathway [26]. The P-IRF3 moves to the nucleus, causing production of IFN-α/β and ISGs. The present study confirmed that the expression of TANK, P-IRF3 and IFN-β proteins were increased by curcumol treatment in the EMCV infected cells. It does appear that curcumol increased the expression and amount of IFN-β and several precursors in the pathway. In order to investigate whether curcumol can directly activate TANK, the protein binding assay of curcumin with TANK and other proteins can be performed.

## Conclusion

EMCV infection of HEK-293 T cells significantly decreased the expression of TANK and P-IRF3 protein in the IFN-β signaling pathway. Curcumol displays anti-EMCV activity by maintaining normal cell morphology and reducing viral load of EMCV infected HEK-293 T cells. We have shown that curcumol appears to upregulate production of IFN-β and this might explain the lack of viral related damage in the infected cells. We conclude that TANK might be the potential targets for the anti-EMCV activity of curcumol. These findings provide new insights into the mechanisms of and new strategies for regulating the infection of EMCV.

## Methods

### Cell lines, viruses, plasmid, compounds and antibodies

HEK-293 T cells was preserved in our laboratory. Cells were cultured and passed in Dulbecco’s modified eagle’s medium (DMEM, Hyclone, USA) containing 10% fetal bovine serum (FBS, BI, Israel) (10% DMEM) and maintenance in DMEM containing 2% FBS (2% DMEM).

The strain of EMCV NJ08 (GenBank: HM641897) was gifted by Professor Jiang Ping of Nanjing Agricultural University. Virus was replicated and harvest in BHK-21 cells infected with EMCV. The titer of 10^8.5^ TCID_50_/ mL was determined by MTT according to the method of Reed-Muench [35].

The recombinant plasmid for the EMCV 3D gene was preserved in the laboratory [36].

Curcumol and ribavirin were purchased from China Food and Drug Control Institute, with 99.9 and 100% purity, respectively. The dissolution of curcumol in DMEM requires 1% DMSO as a co-solvent, while ribavirin is directly dissolved in DMEM.

MDA5 and MAVS Rabbit Polyclonal antibody, GAPDH Mouse Monoclonal antibody, Goat anti-Mouse and Goat anti-Rabbit secondary antibodies were purchased from Proteintech Biotechnology Co., Ltd. (China), TANK Rabbit Polyclonal antibody and IRF3 Rabbit Monoclonal antibody from Abcam (USA); and Phospho-IRF3 Ribbit Polyclonal antibody from Bioss (China).

### Cytotoxicity assay

The cells were treated with different concentration of curcumol (0.1, 0.05, 0.025, 0.0125, 0.00625, 0.003125, 0.0015625, 0.00078125 mg/mL) and ribavirin (4, 2, 1, 0.5, 0.25, 0.125, 0.0625, 0.03125 mg/mL) in the 96-well plates (0.1 mL/well). At the same time, untreated group (no drug) was applied and were incubated at 37 °C and 5% CO_2_ for 72 h. The morphology of cells in the culture was observed every day and photographed. After 72 h of incubation, the supernatant was discarded, 0.02 mL of 3-(4,5-dimethyl-2-thiazolyl)-2,5-diphenyl-2-H- tetrazolium bromide (MTT, Solarbio, China) was added to each well and incubated for 4 h. Subsequently, the supernatant was discarded and 0.15 mL of DMSO was added to each well, and incubated for 30 min. The optical density (OD value) was measured at 490 nm using the plate type multi-function analyzer (Tristar 2 SLB 942, Berthold Technologies, Berthold Technologies). The cytotoxic ratio (CR) was expressed as CR = (OD control—OD test)/OD control. CC_50_ was calculated by nonlinear regression analysis using GraphPad Prism™ software 5.0 (GraphPad Software, Inc. California, USA) [37]. CC_50_ is the constituent concentration at which 50% of cells have developed lesions while MNTC is a maximal concentration of a constituent that enables at least 80% of cells to survive [38].

### Antiviral assay

The HEK-293 T cells were cultured in 96-well plates, infected with 100 TCID_50_/mL EMCV (0.1 mL/well) and allowed to adsorb for 1.5 h [39]. The supernatant was discarded and MNTC of the test compound was serially diluted (2 folds) with DMEM containing 2% FBS into 8 gradients and added to 96-well plates (0.1 mL/well) with 8 replicates per gradient. Meanwhile, uninfected group (no virus and no drug), infected group (with virus, no drug) and ribavirin positive control group (with virus and ribavirin) were applied. The plates were incubated at 37 °C and 5% CO_2_ for 24 h. The inhibition ratio (IR) was calculated as IR = (OD test—OD model) / (OD control—OD model). EC_50_ was calculated using GraphPad Prism™ software 5.0 (GraphPad Software, Inc. California, USA). EC_50_ refers to a compound concentration that is effective in inhibiting 50% of cells infected with EMCV, while selection index (SI) is calculated as SI = CC_50_/EC_50_.

### RNA extraction and quantitative real-time PCR

The HEK-293 T cells were cultured in 6-well plates, infected with 100 TCID_50_/mL EMCV (2 mL/well) and allowed to adsorb for 1.5 h. The supernatant was then discarded and the MNTC of curcumol with 2 folds dilution (0.025, 0.0125, 0.00625 mg/mL) were added to 6 well plates. Meanwhile, uninfected group, infected group and ribavirin positive control group were applied and incubated for 24 h. Total RNA was extracted from the samples, according to the Trizol protocol (Invitrogen, Carlsbad, CA, USA). The RNA concentration and purity were evaluated by using Eppendorf BioPhotometer D30 (Eppendorf, USA). The complementary DNA was synthesized with PrimeScript® RT Master Mix kit with gDNA Eraser (TaKaRa, Dalian, China) according to the manufacturer’s protocol. RT-qPCR was performed by using a 7500 Real Time PCR System (ABI, USA). Relative RT-qPCR was applied to detect the mRNA expression of IFN-β using the 2 × SYBR Green qPCR Master Mix (Low ROX, Biotool, USA). Relative expression levels were determined with the 2^−∆∆Ct^ method. Absolute RT-qPCR was applied to determine EMCV 3D gene, a standard curve was generated using serially diluted plasmid containing 3D gene. The primer sequence for EMCV 3D gene is as: F 5’-TTAGGGCGGGTTTGTAT-3’, R 5’-TTTGTTAGCGGGAGTTA-3’. IFN-β F 5’-ATGACCAACAAGTGTCTCCTCC-3’ R 5’-GCTCATGGAA AGAGCTGTAGTG-3’ And β-actin F 5’-CTGAGCTGCGTTTTACACCC-3’ R 5’-CGCCTTCACCGTTCCAG TTT-3’.

### ELISA for detection of IFN-β production

HEK-293 T cells were cultured in 12-well plates, diluted virus stock solution (100 TCID_50_/mL) was added (1 mL/well) and was allowed for adsorption for 1.5 h. The supernatant was discarded, MNTC of the test compounds with 2 folds dilution (3 concentration gradients) were added to 12 well plate. The cell control and infected group were applied and incubated at 37 °C and 5% CO_2_ for 24 h.

The expression of IFN-β in cell supernatants were detected using enzyme-linked immunosorbent assay (ELISA). Reconstitute the standard with 1.0 mL of sample diluent, and the stock solution with a concentration of 2000 pg/mL. Use the stock solution to produce a twofold dilution series, the undiluted standard is used as the high standard (2000 pg/mL), and the sample diluent is used as the zero standard (0 pg/mL). Add 100 μL of standard and sample per well in coated assay plate, and incubate for at 37 °C 2 h. Remove the liquid of each well. Add 100 μL of biotin-antibody (1x) to each well. And incubate at 37 °C for 1 h. Aspirate each well and wash by filling each well with wash buffer (200 μL), repeat the process 3 times. Add 100 μL of HRP-avidin (1x) to each well, and incubate at 37 °C for 1 h. Repeat the wash process for 5 times. Add 90 μL of TMB substrate to each well, and incubate at 37 °C for 30 min. Add 50 μL of stop solution to each well. The optical density of each well has been measured within 5 min, using a manufacturer’s instruction (Cusabio, China) set to 450 nm. The ELISA results were calculated by Curve Expert software.

### Western blot analysis

The HEK-293 T cells were cultured in 6-well plates, infected with 100 TCID_50_/mL EMCV (2 mL/well) and allowed to adsorb for 1.5 h. The supernatant was discarded and the MNTC curcumol with 2 folds dilution (3 concentration gradients) were added to 6 well plates. The cell control and infected group were applied and incubated at 37 °C and 5% CO_2_ for 24 h. The cells were lysed with RIPA buffer containing 1 mM protease inhibitor, 1 mM phosphatase inhibitor and cells were collected using a cell scraper. Total cell protein was extracted and protein concentration was determined using the BCA protein assay kit (Beyotime Biotechnology, Jiangsu, China). Equal amount of cell lysate was separated on a 10% SDS–polyacrylamide gel and transferred to a polyvinylidene fluoride (PVDF) membrane. Then the membrane was blocked with Tris-buffered Tween 20 (TBST) with 5% non-fat dry milk at 25 °C for 2 h. Next, the membrane was incubated with following primary antibodies overnight at 4 °C: GAPDH Mouse Monoclonal antibody (1:10,000), MDA5 and MAVS Rabbit Polyclonal antibody (1:2000), TANK Rabbit Polyclonal antibody (1:1000), IRF3 Rabbit Monoclonal antibody (1:2000), Phospho-IRF3 Ribbit Polyclonal antibody (1:2000). Then, the membrane was washed with TBST three times, and incubated with the membrane with goat anti-mouse and goat anti-rabbit secondary antibodies (1:20,000) at 25 °C for 2 h. Finally, the target protein was detected by an enhanced chemiluminescence system (Boster, China). Densitometric values of protein bands were quantified by the Image J.

### Statistical analysis

All data were presented as Mean ± SD and repeated at least three times. Data were analyzed using GraphPad Prism™ software 5.0 (GraphPad Software, Inc. California, USA). One-way analysis of variance (ANOVA) followed by a Dunnett’s post-test was used to determine the difference between the groups, * *P* < 0.05, ** *P* < 0.01, *** *P* < 0.001.

## Supplementary Information

**Additional file 1.**

## Data Availability

The datasets used and analysed during the current study are available from the corresponding author on reasonable request.

## Abbreviations

CC_50_: 50% Cytotoxic concentration
CR: Cytotoxic ratio
DMEM: Dulbecco’s modified eagle’s medium
DMSO: Dimethyl sulfoxide
EMCV: Encephalomyocarditis virus
HEK-293 T: Human embryonic kidney 293 T
IRF3: Interferon regulatory Factor 3
IKK-ε: IKB kinases
IFN-β: Interferon-β
MTT: 3-(4,5-Dimethyl-2-thiazolyl) -2,5-diphenyl-2-H-tetrazolium bromide
MDA5: Melanoma differentiation- associated protein 5
MAVS: Mitochondrial antiviral signaling protein
MX: Myxovirus resistance protein
MNTC: Maximum non-toxic concentration
MIR: Maximum inhibition ratio
OASs: 2’-5’ Oligoadenylates synthesis
PKR: Double-stranded RNA-dependent protein kinase
PKR: Double-stranded RNA-dependent protein kinase
SI: Selection index
TBK1: TANK-binding kinase 1
TANK: TRAF family member-associated NF-κB activator
TCID_50_: 50% Tissue culture infective does
