# Supplementary material for: Curcumol inhibits encephalomyocarditis virus by promoting IFN-β secretion
Source: BMC Vet Res. 2021 Sep 30;17:318. doi: 10.1186/s12917-021-03015-4 (PMC8482695; doi:10.1186/s12917-021-03015-4)
Supplement: Supplementary file 1 — Additional file 1. [file 12917_2021_3015_MOESM1_ESM.docx]

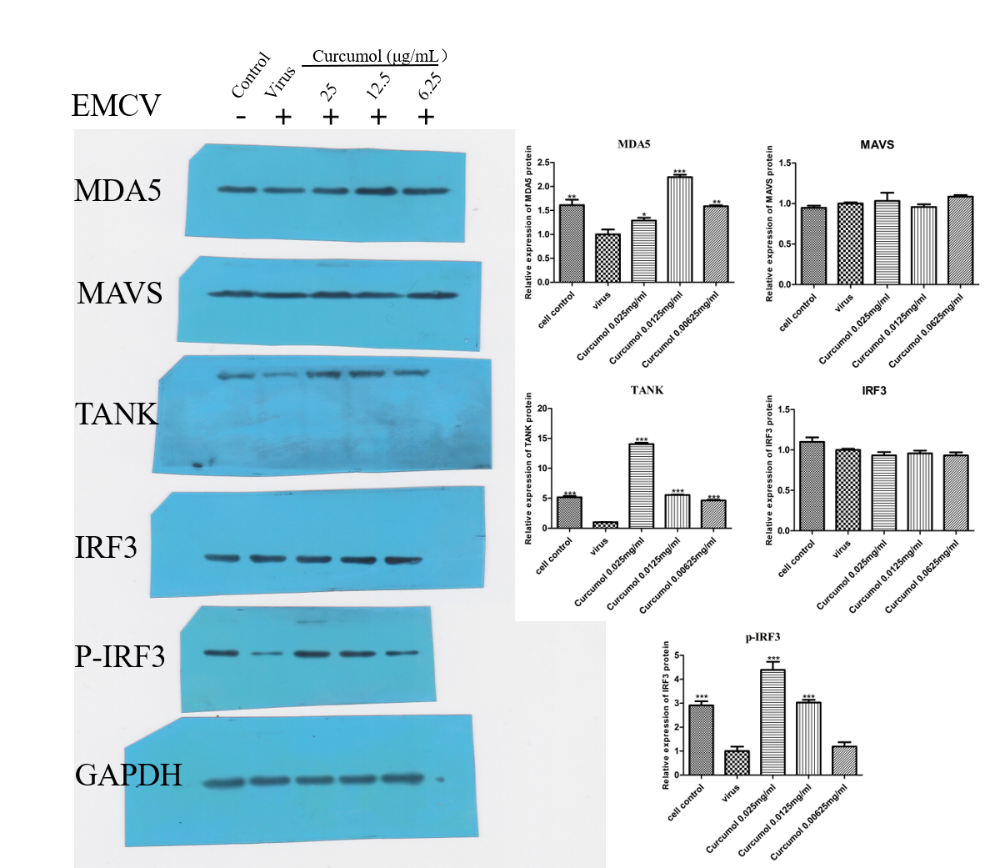


Figure 4. Curcumol (0.025, 0.0125 and 0.00625 mg/mL) and ribavirin (0.25, 0.125 and 0.0625 mg/mL) were selected to treat EMCV-infected HEK-293T cells for 24 h, and the expression of MDA5, MAVS, TANK, IRF3, P-IRF3 protein were detected by Western blot. Densitometric values of protein bands were quantified by the Image J. Data were analyzed using GraphPad Prism^TM^ software 5.0 (GraphPad Software, Inc. California, USA). One-way analysis of variance (ANOVA) followed by a Dunnett’s post-test was used to determine the difference between the groups. All groups are compared with EMCV-infected group (* *P* < 0.05, ** *P* < 0.01, *** *P* < 0.001).
